# Occupational Psychosocial Factors in Primary Care Continuing Care Staff

**DOI:** 10.3390/ijerph17186791

**Published:** 2020-09-17

**Authors:** Javier Guerrero Fonseca, Carmen Romo-Barrientos, Juan José Criado-Álvarez, Jaime González-González, José Luis Martín-Conty, Alicia Mohedano-Moriano, Antonio Viñuela

**Affiliations:** 1 Occupational Risk Prevention Service, Management of Integrated Care of Talavera de la Reina, Castilla-La Mancha Health Service (SESCAM), 45600 Talavera de la Reina (Toledo), Spain; jagufo@sescam.jccm.es; 2 Mental Health Service, Management of Integrated Care of Talavera de la Reina, Castilla-La Mancha Health Service (SESCAM), 45600 Talavera de la Reina (Toledo), Spain; mcromo@sescam.jccm.es; 3 Department of Medical Sciences, Faculty of Health Sciences, University of Castilla-La Mancha (UCLM), 45600 Talavera de la Reina (Toledo), Spain; jaimeg@sescam.jccm.es (J.G.-G.); Alicia.Mohedano@uclm.es (A.M.-M.); 4 Institute of Health Sciences of Castilla-La Mancha, Ministry of Health, 45600 Talavera de la Reina (Toledo), Spain; 5 Santa Olalla Health Center, Management of Integrated Care of Talavera de la Reina, Castilla-La Mancha Health Service (SESCAM), 45600 Talavera de la Reina (Toledo), Spain; 6 Nursing Department, Faculty of Health Sciences, University of Castilla-La Mancha (UCLM), 45600 Talavera de la Reina (Toledo), Spain; joseluis.martinconty@uclm.es (J.L.M.-C.); antonio.vinuela@uclm.es (A.V.)

**Keywords:** psychosocial risk, health personnel, primary health care, occupational health, emergency professional, emotional exhaustion

## Abstract

This involves studying the psychosocial factors among the emergencies staff of primary care and seeing if there are differences with the primary health care staff at the Primary Care of the Integrated Care Management of Talavera de la Reina (Spain). Descriptive epidemiological study of type transversal. They have participated 51 emergencies staff of primary care and 50 primary health professionals from a sample of urban and rural health centres. The F-Psico 3.1 questionnaire has been used to evaluate the nine psychosocial risk factors. The emergencies staff quantify the psychosocial factors of working time (19.6 SD 5.7) and autonomy (69.8 SD 23.2) as a higher risk situation compared to the other health care staff with 3.7 SD 4, 7 and 52.1 SD 21.8, respectively (*p* < 0.05). In addition, the role performance is valued as a lower risk situation by the emergencies staff of primary care (*p* < 0.05). The workload assessment is the only difference between the emergencies staff of primary care in urban centres (61.5 SD 17.6) and rural (45.2 SD 18.4) (*p* < 0.05). Women have the highest workload (*p* < 0.05). It is necessary to apply preventive measures and policies applicable to women who work in emergencies, especially in urban areas to reduce their workload.

## 1. Introduction

The psychosocial factors concept was defined by the joint International Labour Organisation (ILO/WHO, Geneva, Switzerland) in 1984 as “those conditions present in a work situation directly related to the organisation of work and their social environment, with the content of work and the completion of the task, and that are presented with the capacity to affect the development of work and health (physical, psychic or social of the worker” [1,2,3,4]. Exposure to adverse psychosocial risk factors in the workplace leads to distress and diseases which, if they continue, increase the rates of absenteeism, work conflicts, workers leaving jobs, and psychosomatic pathology [5,6,7]. The results of a work-person interaction can be positive if the person is given the chance to develop his/her capacities. Thus, one characteristic that differentiates psychosocial factors from other work conditions is that, albeit being potential risk factors, a preventive objective is not to eliminate or reduce them, but to optimise them to avoid adverse effects and to promote their beneficial effects [1,5,6,7,8,9]. Psychosocial factors can affect motivation and satisfaction with work, and may generate stress, depending on how workers perceive them, and their capacities to face them and respond to them [1,5,7]. Therefore, the psychosocial reality refers to the objective conditions of the workplace and those that workers have withstood and experienced. Psychosocial risks and stress are becoming priority lines of action in health [1,2,5,6,10]. Healthcare workers are one of the groups most affected by their psychosocial working environment [5,11] because they present more signs of occupational psychiatric morbidity than other workers [1,3,6,12,13].

In 2005, the Castilla-La Mancha Health Service (SESCAM) created the Emergencies Staff of Primary Care (ESPC, PEAC in Spanish) figure to replace the support figure (Spanish Decree 63/2005), made up of doctors and nurses who render their services to (urban and rural) primary healthcare centres during working hours that cannot be covered by Primary Healthcare (PH) team professionals as it would mean they work beyond maximum work schedules and would not enjoy legally established rest periods; this was preferentially applied to a basic health area; that is, for PH working hours with 1500 h worked a year. These staff members work in outpatients PH departments and their contract is of a temporary or stand-in kind. In such departments, a relation has been observed between stress caused by work shifts, age, smoking habit, living with one’s partner, and more year’s work experience on the job [1,12]. PH staff [5,6,14] members are characterised by many physical and psychological demands, a mean control on the job post, and unfavourable social support. This working population tends to suffer occupational stress due to work shifts, as well as problems to conciliate social-family life [3,8,13]. Burnout and professional exhaustion in PH, hospitals and PH services have been studied and measured [10,11,15], as have psychosocial factors in hospital services [16,17], but not in PH [4,9,18]. Identifying the psychosocial risk factors to which health PH professionals may be exposed will allow preventive measures to be taken that can be useful to improve their health and quality of life [1,3,5,6,13,17].

This work aims to study the psychosocial factors in ESPC staff and observe if any differences exist between PH staff with permanent or temporary work contacts (statutory or civil servants) who correspond to the Integrated Healthcare Management in Talavera de la Reina (Spain) of SESCAM.

## 2. Materials and Methods

This work was conducted according to the Health and Safety Committee of the Integrated Healthcare Management in Talavera de la Reina (GAITA) of SESCAM in 2017 and 2018. It is a cross-sectional epidemiological study conducted with all staff (doctors and nurses) of GAITA (SGAITA) in the 15 Health Centres and Integrated Continuous Healthcare Point (ICHP). Health care in the city of Talavera de la Reina and the towns of the area that it covers (75% of the population of GAITA is covered) is offered in five health centres (urban) from 08:00–21:00 h. ICHP is the only healthcare centre for area services in Talavera de la Reina and the area it covers. The remaining GAITA population is attended to in 12 health centres (rural) from 08:00–15:00 h. Beyond this schedule, these centres become continuous or urgent healthcare points.

A population of non-ESPC workers was also studied. To do so, random cluster sampling was done by differentiating between urban and rural. Two urban and three rural health centres were obtained, and all their workers participated (doctors and nurses).

A questionnaire with demographic and occupational variables was used with the ESPC. A decision was made to not use this questionnaire with the non-ESPC workers to maintain their anonymity. To assess the psychosocial risk factors, the F-Psico 3.1 questionnaire was used, which was designed by the National Institute of Health and Hygiene in the Workplace, which has been validated in Spain with a Cronbach’s alpha of 0.895 [7,19,20,21]. This questionnaire has 44 questions that evaluate the nine considered psychosocial risk factors on a 5-point Likert scale: working hours (WH, how occupational activity is arranged and ordered in time terms throughout the week and on every weekday. It assesses the impact of WH on rest periods that working activity allows, their quantity and quality, and the effect that WH have on social life); autonomy (AU, the working conditions referring to workers’ individual capacity and possibility to manage and make decisions about both temporary structuring aspects of their working activity and matters affecting work procedure and organisation); work load (WL, the work demand level that workers face); psychological demands (SD, the various demands that workers face at work), work variety/content (VC, workers feel that their work is meaningful and useful, and goes beyond economic pay); participation/supervision (PS, this is “participation” to explore workers’ engagement in their work and organisation, and “supervision” is workers’ assessment of the degree of control that their superiors have on certain aspects of their work performance); interest in workers/compensation (WTC, the extent to which a company shows its concern about workers’ personal character in the long term); role playing (RP, the tasks corresponding to the job post in terms of its clarity, conflicts or extra burdens); relationships/social support (SRS, relationships established among people in the workplace).

The method presents different numerical values in the evaluation made of each psychosocial factor and a scheduling process to transform these direct scores into risk levels by comparing them with pre-set percentiles. Scheduling risk levels is done with accordance with the pre-set percentiles in F-Psico 3.1: Percentile ≥ P85 a very high risk; P75 ≤ Percentile < 85 a high risk; P65 ≤ Percentile < 75 a moderate risk; Percentile < 65 a suitable situation [19,20].

In order to apply the instrument, no explicit consent needs to be acquired from participants because they voluntarily and anonymously participate, as indicated in the information administered to workers. For the application of the instrument, the explicit consent of the participants is not necessary for participating voluntarily and anonymously, as indicated in the information administered to workers. The questionnaire was self-administered, and the answers were collected by internal post to maintain data anonymity.

The descriptive and inferential statistical analysis was done using the parameters according to the variable’s scale, we used the ANOVA test to compare quantitative variables or the Chi-square test or Fisher’s exact test to compare qualitative variables, such as the gender or professional categories, contract type or years of work experience. A 95% confidence level was set. The SPSS statistical package for Windows was used for the data analysis (Statistical Package Social Sciences, version 15.0, IBM, Armonk, NY, USA), along with the F-Psico 3.1 software [19].

## 3. Results

The F-Psico 3.1 questionnaire was sent to 118 workers, of whom 68 (57.6%) belonged to ESPC, and 51 answered (75% participation rate). Of the PH staff, all the workers (100%) who received the questionnaire answered it (n: 50).

The ESPC members included 33 females (64.7%) and 18 males (35.3%), whose mean age for males was 49.3 SD 11.4 years (Median: 44; Range: 27–68), and 42.7 SD 8.6 years (Median: 40; Range: 27–62) for females. Among the ESPC members, holding a stand-in contract predominated with 42 workers (82.4%), while the remaining nine (17.6%) had a temporary contract. The following professional categories were found: 26 doctors (51%) with 21.7 SD 8.7 years work experience (mean age: 51.3 SD 9.6 years), and 25 female nurses (49%) with 14.5 SD 4.6 years work experience (mean age: 39.1 SD 6.3 years) (*p* < 0.05). No statistically significant differences (*p* > 0.05) appeared for the gender of the professional categories, contract type or years’ work experience.

The ESPC worked 1,826 SD 391.94 h/year in 2017 and 1700.6 SD 444.2 h/year in 2018, with no statistically significant differences (*p* > 0.05). No differences appeared in the annual count of worked hours between males and females, although females worked fewer mean hours in 2017 and 2018 with 109.6 and 186.2 h, respectively. In the rural health centres, the mean number of hours they worked a year was bigger, with 213.3 and 219.6 h more in 2017 and 2018, respectively, but with no statistically significant differences (*p* > 0.05).

Among the psychosocial factors included in the F-Psico questionnaire used with the ESPC per gender (Table 1), the work demand level (measured as WL) that females had to face was 11.2 points higher than that for males, with a statistically significant difference (*p* < 0.05). For the other psychosocial factors, no statistically significant differences were found (*p* > 0.05) between the male and female ESPC members. No differences were observed for professional categories (doctors and nurses).

In rural health centres, there were 37 (72.5%) professionals and 14 in the urban areas (27.5%) (Table 2). The mean number of years worked was 19.4 SD 7.8 years, as opposed to 15 SD 7.1 of urban health centres (*p* < 0.05). The WL section was the only one with statistically significant differences (*p* < 0.05) between the ESPC members from urban and rural health centres (Table 2). The professionals from urban areas assessed this factor with 61.5 SD 17.6 points (Median: 62.5; Range: 36–83), while those from rural areas did so with 45.2 SD 18.4 points (Median: 43; Range: 17–89) for WL. In urban areas, females evaluated WL with higher values, with 63.2 SD 16.4 points vs. 48.8 SD 20.7 for rural areas, with statistically significant differences (*p* < 0.05). No statistically significant differences (*p* > 0.05) appeared for males according to health centres, although a difference of 15.2 points was found between urban (55 SD 24.24 points) and rural (39.8 SD 13.4 points) areas.

Table 3 shows which different psychosocial factors obtained statistically significant differences (*p* < 0.05) between ESPC and the remaining PH staff. The ESPC members quantified the WH factor on their rest periods and their social life as a higher risk situation than the other PH staff members with a difference of 15.9 points (*p* < 0.05), and gave an evaluation of 19.6 SD 5.7 points in the working hours (WH) section. Moreover, 58% considered WH a suitable situation, as opposed to 100% of the other staff members. The ESPC members obtained 26 points (Range: 9–33), while the other staff members obtained 3.7 SD 4.7 points, with a results range of 0–17 points. No statistically significant differences (*p* > 0.05) were found for WL. The ESPC members identified the “Autonomy (AU)” factor as a high or very high-risk situation (42%) compared to the other PH staff members (6%), with statistically significant differences (*p* < 0.05). The definition of the job post as role Playing (RP) obtained a higher score with 58.1 points by the PH staff (70% believed it to be a high or a very high risk situation), as opposed to the 46.4 points that the ESPC members obtained (55% considered it a high or a very high risk situation) (*p* < 0.05).

## 4. Discussion

The females holding a work contract as ESPC workers in the urban GAITA area stated having a heavier WL, and a difference of up to 23 points was found between ESPC workers and males working in rural areas. These gender differences could be due to organisation and type of work done because the ESPC members work a mean of 2 or 3 shifts a week lasting 17 or 24 h (weekends). These shifts alter their personal and family lives. The PH professionals (who do not work such shifts) obtained higher values in this section than the ESPC workers, but no statistically significant differences appeared (*p* > 0.05). Therefore, it is reasonable to assume that these differences were due to the type of work performed, and to family-working life conciliation difficulties. Several works have reported that female healthcare workers [10,14,15], particularly nurses, present higher levels of stress, face more psychological demands at work, and receive less social and leadership support [5,13,15,22]. No significant differences were herein found in professional categories. Females indicated lower self-compassion levels, which could explain some of the differences found [23,24]. Females represented 65% of the workers, which must be considered by managers and planners to start implementing corrective and preventive measures [2,12,13,14,15,22].

Psychosocial risks not only make workers’ quality of life worse but can also lead to burnout and diseases if not properly managed [3,5,6,13,25]. PH work presents changing characteristics that mean that professionals must constantly adapt, which increases occupational stress [5,6,8,9,10,11,18,26]. Quantifying a higher risk situation for WH and quantifying the RP factor less by ESPC members can lead to different problems. Nonetheless compared to other healthcare professionals, ESPC personnel quantify both autonomy (AU) (*p* < 0.05) and relationships/social support (RSS) (*p* > 0.05) as higher risk situations, while psychological demands (PD) come over as a lower risk situation (*p* > 0.05) [1,3,13]. The fact that ESPC or PH staff tend to be younger than other healthcare professionals could explain why these professionals wish more autonomy to do their job [5,6,27].

The differences between urban and rural areas can be explained by the distinct extra healthcare burden experienced by PH personnel [17,18]. In rural areas, fewer people go to PH because PH medicine/nursing consultations are more accessible and due to healthcare longitudinally. These situations do not occur in urban areas where people may have to wait a few days to be attended. So, some patients go to emergency services. In our case, only one Integrated Continuous Healthcare Point (ICHP) exists in urban areas, which may imply an extra healthcare burden on occasions and, thus, stress and emotional fatigue [13,27], which could explain the differences between urban and rural areas. Another possibility is that as healthcare staff’s job posts remain longer in employment exchanges, they may opt for rural areas with less WL, which might explain why these staff members are older than those in urban areas [4,11,12,18].

Some healthcare professionals working for hierarchised healthcare institutions may often feel poorly engaged, insufficiently acknowledged, perform badly defined tasks, face no internal promotion prospects, and lack management’s support [6,10,16,26]. In our case, however, no differences were found for interest in workers/compensation (WTC) or them participating in and supervising management (PS), which contributes to workers’ psychosocial protection. Those healthcare workers with higher depersonalisation indices perceive their work is not suitably valued by patients, family relations, colleagues, and superiors [3,13]. Believing that one’s work is useful and well-valued has a protector effect for depersonalisation. Healthcare workers, which is often vocational work, need to feel they take part and form part of the company, and their work is recognised and valued [3,4,8,9,10,11,12,13,18,26].

Stress increases and satisfaction lowers with WL and RP which, in turn, influence interpersonal relationships. Stress is closely related to the quantity of psychosocial resources that one possesses, or believes he possesses, to face workplace demands [3,6,8,13]. An association is known to exist between psychosocial WL and stress symptomatology among healthcare workers [3,5,11,13]. These aspects diminish work performance and blur the context that allows and helps professionals to continue working their profession in a lively and active manner. The likelihood of presenting considerable emotional exhaustion and depersonalisation is higher for the professionals who often meet suffering and death, and their work negatively impacts their family life [22]. The probability of feeling much emotional exhaustion is higher for people with a heavy WL [3,10,13]. Healthcare professionals’ burnout and stress levels have increased in recent years [3,10,13,15], but the psychosocial reality does not only depend on the work conditions or on a country’s socio-economic situation, but on how workers perceive these conditions [3,4,8,11,15,16].

We found practically no differences in the socio-demographic variables (gender, age, professional career) between the ESPC and the other PH professionals. Therefore, the observed differences could be explained by the type of work that each does. One limitation of this work is its cross-sectional design. So, no causality relations can be established. As we obtained a high response rate, the results can be considered valid in the studied population. The participation or response rate was very good as 75% of the ESPC working population participated, as did 100% of the PH professionals in this study, so this population can be considered representative. However, we ought to remember that it is often the most hostile, tired, and vulnerable staff members who do not participate in these studies [10,11,15]. Our data came from self-report questionnaires with their inherent limitations, which could still be more efficient in collecting data and providing subjects with more intimacy than other types of questionnaires or measuring techniques [10,21,26]. Checking our results with other works that have measured psychosocial factors and stress is a complicated task because of the different methodologies and measuring instruments employed [4,5,6,11,16,21,25,26]. F-Psico 3.1 is a questionnaire that loses details when all the possible and risk situations are grouped into a few general factors, but it is the only one that assesses the situation of people who might have a low WL and, therefore, feel little professional self-esteem [21].

## 5. Conclusions

Workers’ psychosocial environment acts as a risk in healthcare activities. It is necessary for healthcare professionals to acquire skills that help them to connect to others, be kind to oneself and observe reality in a level-headed way, and from a perspective that can protect them and reduce their emotional exhaustion. It is very important to pay special attention to the gender differences herein observed. Setting up awareness-raising, training, and adaptation measures is believed to be necessary for lower WL and to avoid physical, psychic, and social consequences.

## Figures and Tables

**Table 1:** Psychosocial factors in Emergency Staff Primary Care (ESPC) workers according to gender.

Psychosocial Factors	Males (*n* = 18)		Females (*n* = 33)		Statistical Significance
	Mean SD	Range	Mean SD	Range	
Working hours (WH)	18.6 SD 6.1	9–27	20.2 SD 5.6	9–33	*p* > 0.05
Autonomy (AU)	62.8 SD 26.3	20–107	73.6 SD 20.8	32–104	*p* > 0.05
Workload (WL)	42.4 SD 15.8	21–83	53.6 SD 20.3	17–89	*p* < 0.05 *
Psychological demands (PD)	67.7 SD 17.7	31–100	67.1 SD 16.6	42–96	*p* > 0.05
Work variety/content (VC)	17.6 SD 10.4	0–38	21.0 SD 10.1	0–42	*p* > 0.05
Participation/Supervision (PS)	49.1 SD 16.1	22–75	45.0 SD 20.6	4–85	*p* > 0.05
Interest in workers/compensation (WTC)	45.5 SD 20.8	5–70	48.2 SD 15.6	12–70	*p* > 0.05
Role playing (RP)	43.1 SD 20.9	1–66	48.2 SD 21.8	1–98	*p* > 0.05
Relationships and social support (RSS)	30.3 SD 22.7	0–65	35.1 SD 17.4	0–62	*p* > 0.05

*n*: number of subjects; SD: standard deviation.

**Table 2:** Psychosocial factors in Emergency Staff Primary Care (ESPC) workers according to the workplace.

Psychosocial Factors	Urban (*n* = 14)		Rural (*n* = 37)		Statistical Significance
	Mean SD	Range	Mean SD	Range	
Working hours (WH)	19.1 SD 6.8	9–33	19.8 SD 5.4	9–29	*p* > 0.05
Autonomy (AU)	77.6 SD 20.8	38–104	66.8 SD 23.6	20–107	*p* > 0.05
Work load (WL)	61.5 SD 17.6	36–83	45.2 SD 18.4	17–89	*p* < 0.05 *
Psychological demands (PD)	68.0 SD 15.9	42–95	67.1 SD 17.3	31–100	*p* > 0.05
Work variety/content (VC)	23.3 SD 11.6	0–42	18.5 SD 9.5	0–38	*p* > 0.05
Participation/Supervision (PS)	44.1 SD 17.2	23–85	47.3 SD 19.9	4–83	*p* > 0.05
Interest in workers/compensation (WTC)	49.9 SD 14.7	12–68	46.2 SD 18.5	5–70	*p* > 0.05
Role playing (RP)	48.6 SD 16.2	16–79	45.6 SD 23.2	1–98	*p* > 0.05
Relationships and social support (RSS)	38.0 SD 15.2	6–61	31.6 SD 20.6	0–65	*p* > 0.05

*n*: number of subjects; SD: standard deviation; ESPC: Emergency Staff Primary Care.

**Table 3:** Quantification of Psychosocial Factors in Emergency Staff Primary Care (ESPC) and healthcare workers.

Psychosocial Factors	ESPC (*n* = 51)						PH (*n* = 50)						Statistical Significance
			Situation or Risk (Percent)						Situation or Risk (Percent)				
	Mean SD	Range	Suitable Situation	Moderate	High	Very High	Mean SD	Range	Suitable Situation	Moderate	High	Very High	
Working hours (WH)	19.6 SD 5.7	9–33	58	22	16	4	3.7 SD 4.7	0–17	100	0	0	0	*p* < 0.05 *
Autonomy (AU)	69.8 SD 23.2	20–107	46	12	22	20	52.1 SD 21.8	0–95	78	16	4	2	*p* < 0.05 *
Work load (WL)	49.6 SD 19.4	17–89	49	14	8	29	52.3 SD 22.3	6–91	44	6	10	40	*p* > 0.05
Psychological demands (PD)	67.3 SD 16.8	31–100	29	27	13	31	69.7 SD 18.6	36–104	34	12	12	42	*p* > 0.05
Work variety/content (VC)	19.7 SD 10.2	0–42	82	12	6	0	23.4 SD 11.1	4–53	76	16	2	6	*p* > 0.05
Participation/Supervision (PS)	46.4 SD 19.1	4–85	12	6	20	62	53.8 SD 19.5	15–87	10	6	4	80	*p* > 0.05
Interest in workers/compensation (WTC)	47.2 SD 17.4	5–70	55	6	14	25	51.3 SD 17.3	2–73	40	10	16	34	*p* > 0.05
Role playing (RP)	46.4 SD 21.4	1–98	33	12	12	43	58.1 SD 22.8	1–101	24	6	4	66	*p* < 0.05 *
Relationships and social support (RSS)	33.3 SD 19.3	0–65	35	12	10	43	30.5 SD 14.8	0–70	44	10	18	28	*p* > 0.05

*n*: number of subjects; SD: standard deviation; ESPC: Emergency Staff Primary Care; PH: Primary Healthcare.
